# Soluble CD146, a cerebrospinal fluid marker for neuroinflammation, promotes blood-brain barrier dysfunction

**DOI:** 10.7150/thno.37142

**Published:** 2020-01-01

**Authors:** Daji Wang, Hongxia Duan, Jing Feng, Jianquan Xiang, Liqun Feng, Dan Liu, Xuehui Chen, Lin Jing, Zheng Liu, Dexi Zhang, Hongjun Hao, Xiyun Yan

**Affiliations:** 1 Savaid Medical School, University of Chinese Academy of Sciences, Beijing, China.; 2 Key Laboratory of Protein and Peptide Pharmaceutical, Institute of Biophysics, Chinese Academy of Sciences, Beijing, China.; 3 Neuroimmunology Laboratory, Peking University First Hospital, Beijing, China.; 4 School of Basic Medical Sciences, Southwest Medical University, Sichuan, China.; 5 Beijing Anzhen Hospital, Capital Medical University, Beijing, China.

**Keywords:** sCD146, cerebrospinal fluid, blood-brain barrier damage and neuroinflammation.

## Abstract

The blood-brain barrier (BBB) dysfunction is an initial event of various neuroinflammatory diseases. However, the absence of reliable markers and mechanisms for BBB damage greatly limits the diagnosis and treatment of neuroinflammatory diseases. Soluble CD146 (sCD146) is mainly derived from vascular endothelial cells (ECs) and highly elevated in inflammatory settings. Based on a small cohort, our previous study showed that sCD146 is elevated in the cerebrospinal fluid (CSF) of multiple sclerosis (MS), which is accompanied with BBB damage. Nevertheless, whether sCD146 monitors and regulates the BBB dysfunction remains unknown.

**Methods:** Coupled serum and CSF samples from patients with or without neuroinflammatory diseases were collected via multicenter collaborations. sCD146 was measured by sandwich ELISA using anti-CD146 antibodies AA1 and AA98, both of which were generated in our laboratory. The correlations between sCD146 and other clinical parameters or inflammatory factors were analyzed by Spearman's correlation coefficient analysis. The role of sCD146 on BBB function was examined in an *in vitro* BBB model.

**Results:** Between July 20, 2011, and February 31, 2017, we collected coupled serum and CSF samples from 823 patients, of which 562 (68.3%) had neuroinflammatory diseases, 44 (5.3%) had remitting MS, and 217 (26.4%) had non-inflammatory neurological diseases (NIND). We found that sCD146 in CSF, but not in serum, is abnormally elevated in neuroinflammatory diseases (37.3 ± 13.3 ng/mL) compared with NIND (4.7 ± 2.9 ng/mL) and remitting MS (4.6 ± 3.5 ng/mL). Abnormally elevated CSF sCD146 is significantly correlated with the hyperpermeability-related clinical parameters of BBB and neuroinflammation-related factors. Moreover, CSF sCD146 shows higher sensitivity and specificity for evaluating BBB damage. Using an *in vitro* BBB model, we found that sCD146 impairs BBB function by promoting BBB permeability via an association with integrin αvβ1. Blocking integrin αvβ1 significantly attenuates sCD146-induced hyperpermeability of the BBB.

**Conclusion:** Our study provides convincing evidence that CSF sCD146 is a sensitive marker of BBB damage and neuroinflammation. Furthermore, sCD146 is actively involved in BBB dysfunction.

## Introduction

The homeostasis of the central nervous system (CNS) microenvironment requires blood-brain barrier (BBB) integrity 1. The quiescent BBB is a physical and functional structure organized by brain endothelial cells (ECs) that are in contact with various CNS cell types, such as pericytes and astrocytes 2. All the components form a multilayered membrane structure and constitute an extremely low-rate permeability barrier. The integrity and low permeability of the BBB are essential for proper neuronal function in the CNS. The BBB allows for the highly selective diffusion of nutrients and separates the CNS from soluble inflammatory mediators and effector immune cells from the peripheral circulation 3. BBB dysfunction contributes to the initiation of many neuroinflammatory diseases, including demyelinating diseases, brain tumors and infections 4, 5. Numerous reports have suggested that inflammatory factors are the major cause of BBB destruction. Under steady-state conditions, inflammatory cytokines are at very low or undetectable levels. However, they are induced rapidly in response to inflammation 6. Pro-inflammatory cytokines play a pivotal role in CNS inflammation through the induction of chemokines and adhesion molecules, followed by the recruitment of immune cells into the parenchyma and the activation of endogenous glial cells. Under CNS inflammation, the tight junctions between brain ECs disappear, and the attached pericytes and astrocytes fall off 7. This damage elevates BBB permeability and leads to further pro-inflammatory factor diffusion and increases the migration of immune cells into the CNS, thereby augmenting diseases. Although reported evidence has shown that BBB dysfunction is associated with progressive neuroinflammation, there is still no effective or reliable marker for clinical diagnosis or to guide treatment due to limited knowledge of the molecular mechanism underlying BBB breakdown.

The adhesion molecule CD146 is primarily expressed at the intercellular junctions of ECs and is reported to be involved in a variety of physiological and pathological processes 8-15. Recently, we reported that CD146 is a mediator that coordinates the interplay of ECs and pericytes to promote BBB development. In neuroinflammatory diseases such as multiple sclerosis (MS), CD146 is upregulated on blood-brain barrier endothelial cells (BBB-ECs) and promotes the transmigration of inflammatory cells into the CNS 16, 17. Furthermore, we found that soluble CD146 (sCD146), the soluble form of membrane CD146, is increased in the cerebrospinal fluid (CSF) of patients with active MS compared to the CSF of patients with inactive MS and that sCD146 positively correlates with the clinical parameters of BBB damage, indicating that sCD146 participates in BBB disruption 18. In addition, numerous reports have shown that an abnormal elevation in serum sCD146 correlates with EC activity and permeability under inflammatory conditions 19, 20. Importantly, in our previous study, we showed that sCD146 promotes inflammation by facilitating the transmigration of immune cells across the BBB 18. However, whether sCD146 directly contributes to BBB dysfunction is still unclear.

In this study, we demonstrate that CSF sCD146 is abnormally elevated in patients with various neuroinflammatory diseases and that the elevation in CSF sCD146 closely correlates with BBB damage. Moreover, we evaluate the function of sCD146 in BBB permeability and provide evidence that sCD146 contributes directly to BBB dysfunction. These findings suggest that CSF sCD146 is a sensitive marker for BBB damage, and that sCD146 is a novel driver of neuroinflammation-BBB disruption.

## Results

### CSF sCD146 is elevated in neuroinflammatory diseases and positively correlated with clinical parameters

**Figure 1:**
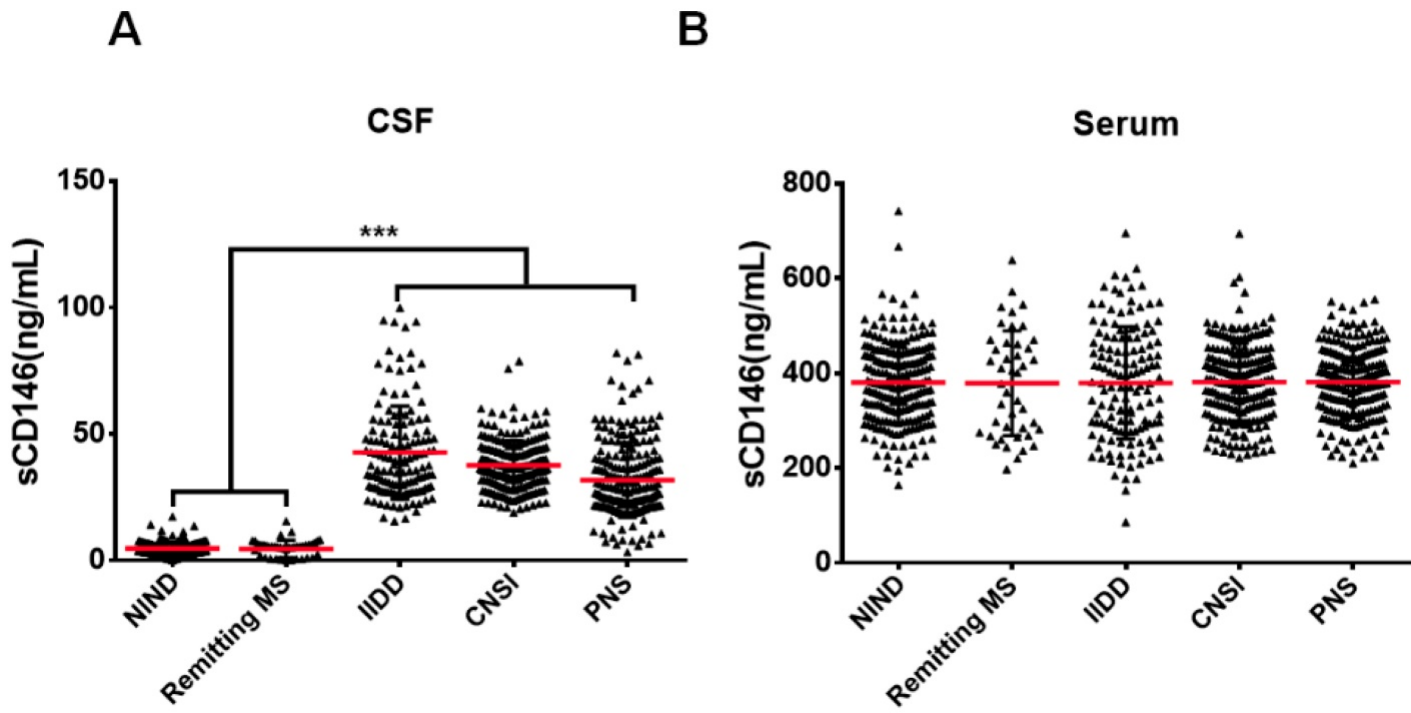
** CSF sCD146 is elevated in neuroinflammatory diseases. (A-B)** sCD146 levels in CSF and serum from patients with NIND (n=217), remitting MS (n=44), IIDD (n=136), CNSI (n=210), PNS (n=216) were assayed using an ELISA sandwich system. *p<0.05; **p<0.01; and ***p<0.001. The data are representative of three independent experiments.

Our previous study showed that sCD146 in the CSF could be used as a marker for disease activity in MS patients 18, which showed obvious BBB damage. To evaluate the role of sCD146 in BBB dysfunction, we enrolled a larger cohort of patients with various neuroinflammatory diseases, which are reported to be associated with BBB damage. We collected coupled CSF and serum samples from 606 patients with neuroinflammatory diseases. The neuroinflammatory diseases included the following: idiopathic inflammatory demyelination diseases (IIDD, n=136), which included relapsing MS (n=93) and neuromyelitis optical spectrum disorders (NMOSD, n=43) 21; CNS infection (CNSI, n=210); peripheral neuropathies (PNS, n=216); and remitting MS (n=44). The patients with IIDD, CNSI or PNS were diagnosed as being in active states with obvious BBB damage, as indicated by MRI results (Figure S1). Coupled CSF and serum samples from 217 patients with non-inflammatory neurological diseases (NIND) were served as controls. Detailed patient information is provided in Table 1. To measure the sCD146 levels in the CSF and serum of these patients, we used an ELISA protocol that was modified from our previous report 18. As shown in Figure 1A-B, the levels of serum sCD146 showed no significant differences between any groups. In contrast, the CSF sCD146 levels in patients with neuroinflammatory diseases were significantly higher than those in patients with NIND (37.3±13.3 ng/mL vs. 4.7±2.9 ng/mL), indicating that an elevated CSF sCD146 level is associated with neuroinflammation. Using samples from patients with NIND, relapsing MS and remitting MS (Table S1), we also confirmed that CSF sCD146 level is positively correlated with the progression of neuroinflammatory diseases, which is consisted with our previous study.

Next, we determined whether CSF sCD146 level is correlated with BBB disruption. In clinical laboratory examinations, several parameters, such as albumin quotient (Q_Alb_), IgG synthesis and myelin basic protein (MBP), are used to evaluate BBB damage for auxiliary diagnosis. To determine the association of CSF sCD146 with BBB damage, we analyzed the correlations of CSF sCD146 with these factors. As shown in Table 2, significantly positive correlations were observed between the level of CSF sCD146 and the level of each parameter, namely, Q_Alb_, IgG synthesis and CSF-MBP, in patients with neuroinflammatory diseases. However, in patients with NIND, CSF sCD146 showed no or mild correlations with these factors. In both non- and neuroinflammatory diseases, CSF sCD146 had no correlations with serum sCD146 or other clinical indexes (age, S-MBP, C-MBP/S-MBP).

### CSF sCD146 is positively correlated with CSF neuroinflammation-related factors

Several CSF neuroinflammation-related factors, including matrix metalloproteinases (MMP2 and MMP9), soluble adhesion molecules (sICAM and sVCAM) and inflammatory factors (IL-1β, IL-17A, IL-10, IFN-γ and TNF-α), have been reported in neuroinflammation and are considered to participate in BBB damage 22-28. In the CSF from healthy subjects, none of or very low levels of these factors are detected, while in the CSF from patients with neuroinflammatory diseases, high levels of these factors are detected and generally indicate the progress of various neuroinflammatory diseases. To further investigate whether CSF sCD146 is associated with BBB damage, we analyzed the relationship between CSF sCD146 and these neuroinflammation-related factors using a CBA kit from 93 patients with IIDD, 109 patients with CNSI and 44 patients with remitting MS. The results showed that the concentrations of CSF MMP2, MMP9, sICAM, sVCAM, IL-1β, IL-17A and IFN-γ were significantly increased in IIDD and CNSI patients compared to remitting MS patients (Table 3). However, TNF-α and IL-10 showed low levels and no significant differences in any of the CSF samples (data not shown).

We further analyzed the correlations between CSF sCD146 and neuroinflammation-related factors. As shown in Table 3, positive correlations between the level of CSF sCD146 and the level of each of MMP2, MMP9, sICAM, sVCAM, IL-1β, IL-17A and IFN-γ were observed in the IIDD and CNSI groups. In contrast, the correlation in remitting MS was nonsignificant.

### CSF sCD146 is sensitive for monitoring BBB damage in neuroinflammatory diseases

**Figure 2:**
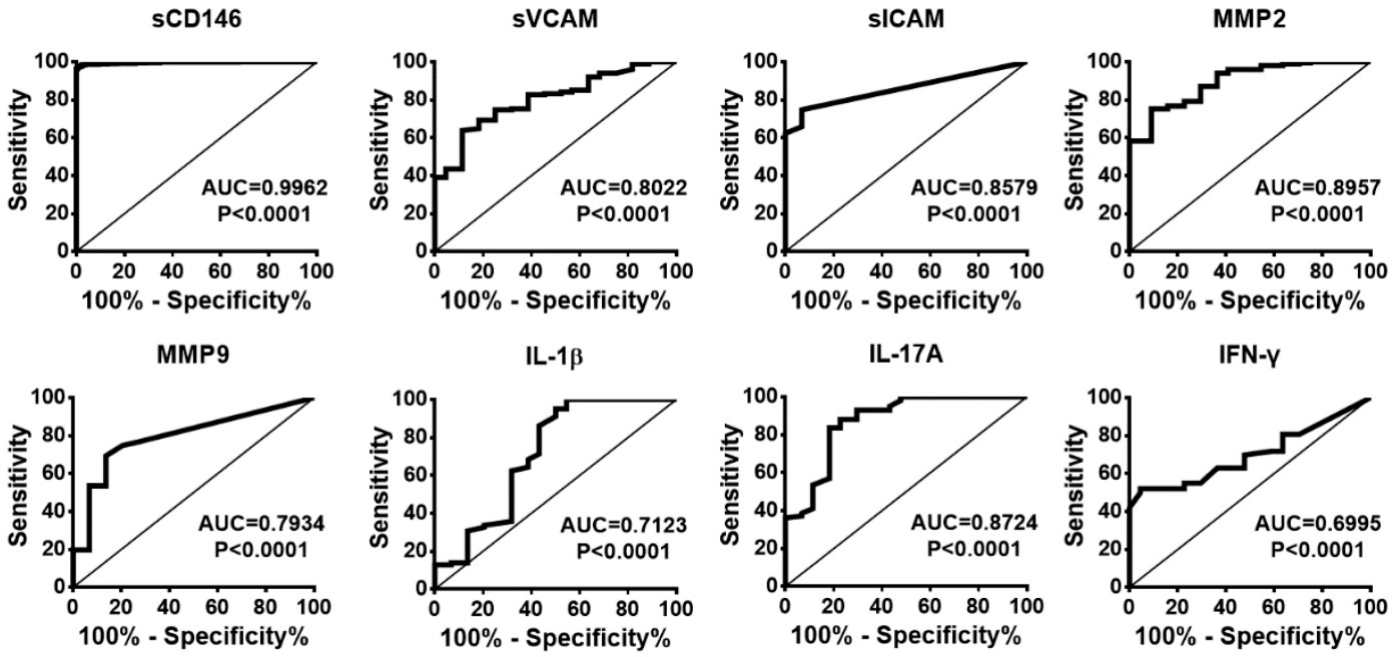
The ROC curves of sCD146 and related molecules to predict the BBB damage.

To evaluate whether CSF sCD146 is preferred for monitoring BBB damage and neuroinflammation, we compared the sensitivity, specificity, positive predictive value (PPV) and negative predictive value (NPV) of CSF sCD146 to other related molecules. Based on the cut-off value of CSF sCD146 (10.5 ng/mL), further analysis showed that the sensitivity of CSF sCD146 was much higher (100%) than those of the other clinical parameters (31.7-73.9%) and the neuroinflammation-related factors (13.3-74.4%). Moreover, we found that CSF sCD146 showed a higher specificity (93.1%), PPV (97.4%) and NPV (100%). Although sVCAM, sICAM and IFN-γ showed 100% specificity and PPV, their NPV (24.6-45.8%) were much lower than that of CSF sCD146 (Table 4). In order to further test the ability of CSF sCD146 in predicting of BBB damage and neuroinflammation, we performed the analysis of receiver operating characteristic (ROC) curve. The ROC curve showed that the area of sCD146 group under the ROC curve is 0.9962, which is much better than other related molecules (Figure 2). In summary, these data demonstrate that sCD146 is sensitive for monitoring BBB damage during the development of neuroinflammatory diseases.

### sCD146 promotes BBB permeability *in vitro*

**Figure 3:**
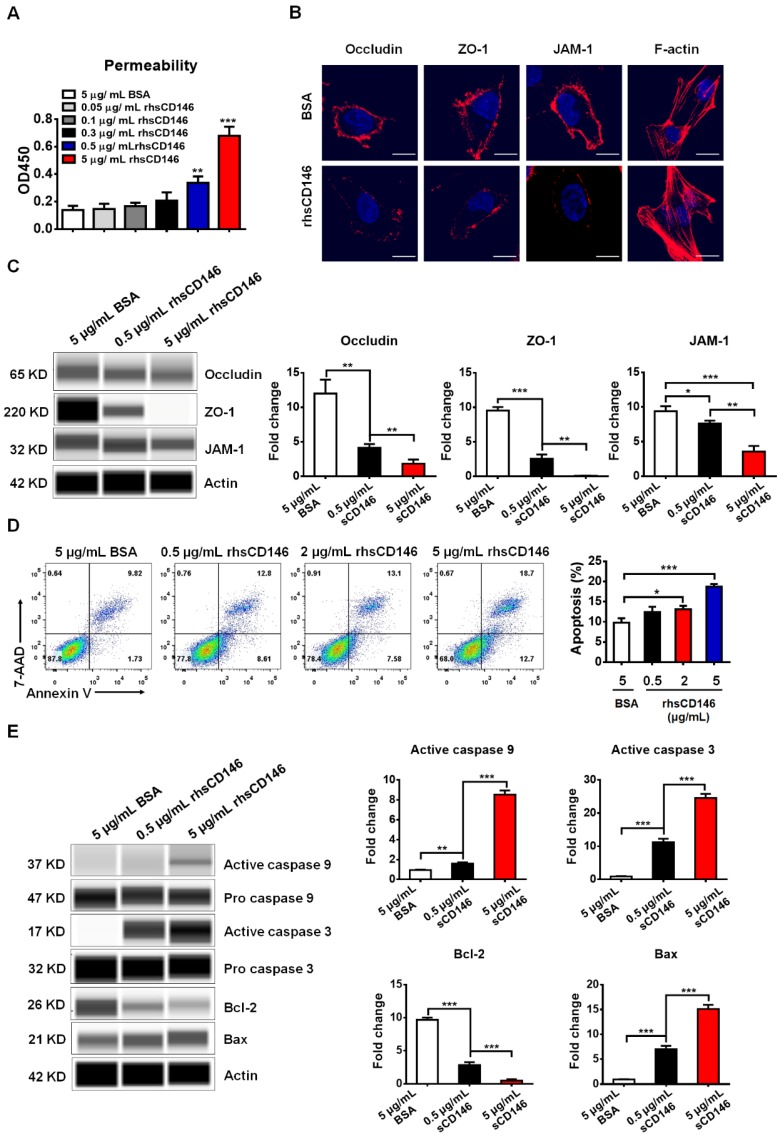
** sCD146 promotes BBB permeability *in vitro*. (A)** Analysis of paracellular barrier function by permeability assay. hCMEC/D_3_ cells were seeded into the upper chambers of a transwell system, and permeability was measured with 0.5 μg/mL HRP after hCMEC/D_3_ cells were incubated with 5 μg/mL BSA, 0.05-5 μg/mL rhsCD146 for 2 h. *p<0.05; **p<0.01; and ***p<0.001. **(B)** Immunofluorescence staining of the TJPs (occludin, ZO-1 and JAM-1) and F-actin after hCMEC/D_3_ cells were treated with 5 μg/mL BSA or rhsCD146 for 4 h. Bar, 10 μm. **(C)** hCMEC/D_3_ cells were preincubated with 5 μg/mL BSA, 0.5 μg/mL or 5 μg/mL rhsCD146, TJP expression levels were verified by western blotting. **(D)** hCMEC/D_3_ cells were treated with 5 μg/mL BSA or 0.5, 2 or 5 μg/mL rhsCD146 for 12 h, and apoptosis was detected by flow cytometry with Annexin V and 7-AAD. **(E)** hCMEC/D_3_ cells were treated with 5 μg/mL BSA, 0.5 rhsCD146 or 5 μg/mL rhsCD146 for 12 h, and cell lysates were used to detect the expression of caspase 9, caspase 3, Bcl-2 and Bax.

Many studies have shown that a high level of serum sCD146 reflects the activity and junction of ECs in different types of cardiovascular diseases 29, 30. Based on these findings, we hypothesized that sCD146 may disrupt the integrity of BBB-ECs. To determine the relationship between sCD146 and BBB disruption, we established an *in vitro* BBB model using hCMEC/D_3_ cells, which has been widely used for evaluating BBB integrity* in vitro* 31. The tightness of the cell confluence was evaluated by crystal violet staining and transendothelial electrical resistance (TEER) (Figure S2 and S3A). After treatment with or without the indicated concentrations of rhsCD146, BBB permeability was evaluated with an HRP tracer. As shown in Figure S4 and Figure 3A, we found that rhsCD146 rapidly binds to unknown receptors of hCMEC/D_3_ cells to promote BBB permeability in a dose-dependent manner. This result is also confirmed by TEER analysis (Figure S3B). The increasing BBB permeability is caused by the loss of tight junctions or the apoptosis of ECs 32, 33. In this *in vitro* BBB model, using immunofluorescence and western blot analysis, we found that treatment with rhsCD146 markedly reduced the expression of cell surface tight junction proteins (TJPs), including occludin, zonula occludens (ZO)-1 and junctional adhesion molecule (JAM)-1 (Figure 3B-C and Figure S5A). Moreover, rhsCD146 treatment induced the reorganization of the actin cytoskeleton to form stress fibers, suggesting the activation of ECs (Figure 3B). In addition, we found that high levels of rhsCD146 significantly promoted the apoptosis of hCMEC/D_3_ cells (Figure 3D). Treatment with rhsCD146 reduced the expression of the anti-apoptosis protein Bcl-2 and increased the expression of the pro-apoptosis protein Bax. Importantly, after rhsCD146 incubation, caspase 9 and caspase 3 were abnormally activated, suggesting that rhsCD146-induced apoptosis of hCMEC/D_3_ cells involves the caspase 9 and caspase 3 pathways (Figure 3E and Figure S5B). In summary, these data suggest that sCD146 increased BBB permeability at least partially by reducing the expression of TJPs and facilitating BBB-ECs apoptosis, indicating that sCD146 is a novel molecule that participates in BBB dysfunction.

### sCD146 interacts with integrin αvβ1

**Figure 4:**
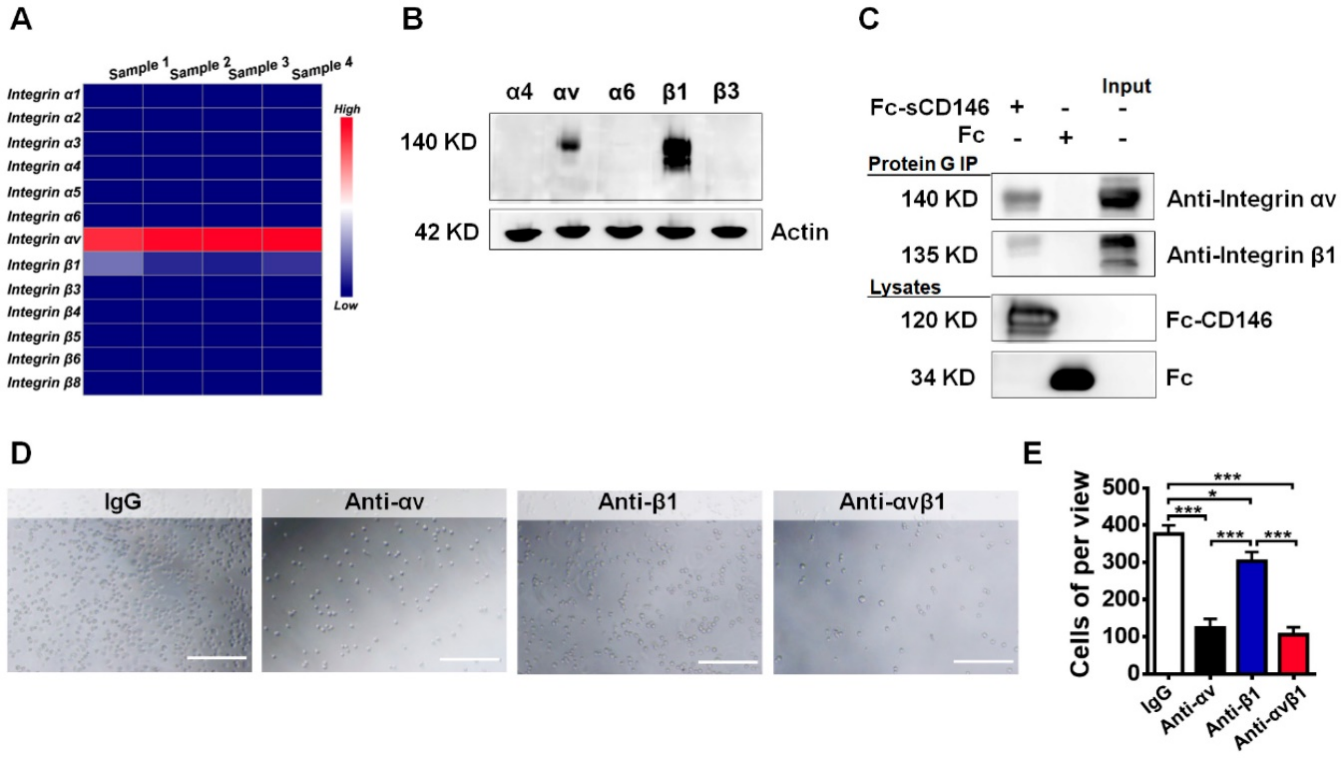
** sCD146 interacts with integrin αvβ1. (A)** Total RNA of hCMEC/D_3_ cells was extracted to measure the mRNA expression of integrin subunits by Q-PCR. **(B)** Whole-cell lysates of hCMEC/D_3_ cells were collected, and the protein expression levels of the integrin subunits were detected by western blot. **(C)** Co-IP assays show the association between sCD146 and integrin αvβ1 in hCMEC/D_3_ cells. Protein levels were analyzed with anti-integrin αv and β1 antibodies. **(D-E)** Cell adhesion assay. The interaction between sCD146 and integrin αvβ1 was blocked by anti-integrin αv and β1 antibodies. Bar, 100 μm. *p<0.05; **p<0.01; and ***p<0.001.

To evaluate how sCD146 promotes BBB permeability, we tried to identify the associated membrane molecules of sCD146 on BBB-ECs. Integrins are reported to play a central role in cell adhesion and blood vessel permeability 34-36. Thus, we used quantitative polymerase chain reaction analyses to confirm the expression of particular integrin subunits in hCMEC/D_3_ cells. We found that the integrin αv and β1 subunits were highly expressed in hCMEC/D_3_ cells, whereas the expression levels of α1, α2, α3, α4, α5, α6, β3, β4, β5, β6 and β8 in hCMEC/D_3_ cells were relatively low (Figure 4A). The primers for the integrins are shown in Table S2. This result was partially confirmed by western blot analysis (Figure 4B).

Next, we evaluated whether sCD146 is associated with the integrin αv and β1 subunits in hCMEC/D_3_ cells. A coimmunoprecipitation (Co-IP) assay showed that integrin αv and β1 were associated with sCD146 in hCMEC/D_3_ cells (Figure 4C). Moreover, the direct interaction between sCD146 and integrin αvβ1 was confirmed by cell adhesion assay 37. As shown in Figure 4D-E, pretreatment of hCMEC/D_3_ cells with neutralizing antibodies for αv or β1 significantly reduced the number of adherent cells in the presence of sCD146, and the reduction was more obvious with the combination of anti-αv and anti-β1 antibodies.

### MAPK, Akt and NF-кB signaling pathways are involved in sCD146-integrin αvβ1 induced-hyperpermeability of hCMEC/D_3_ cells

**Figure 5:**
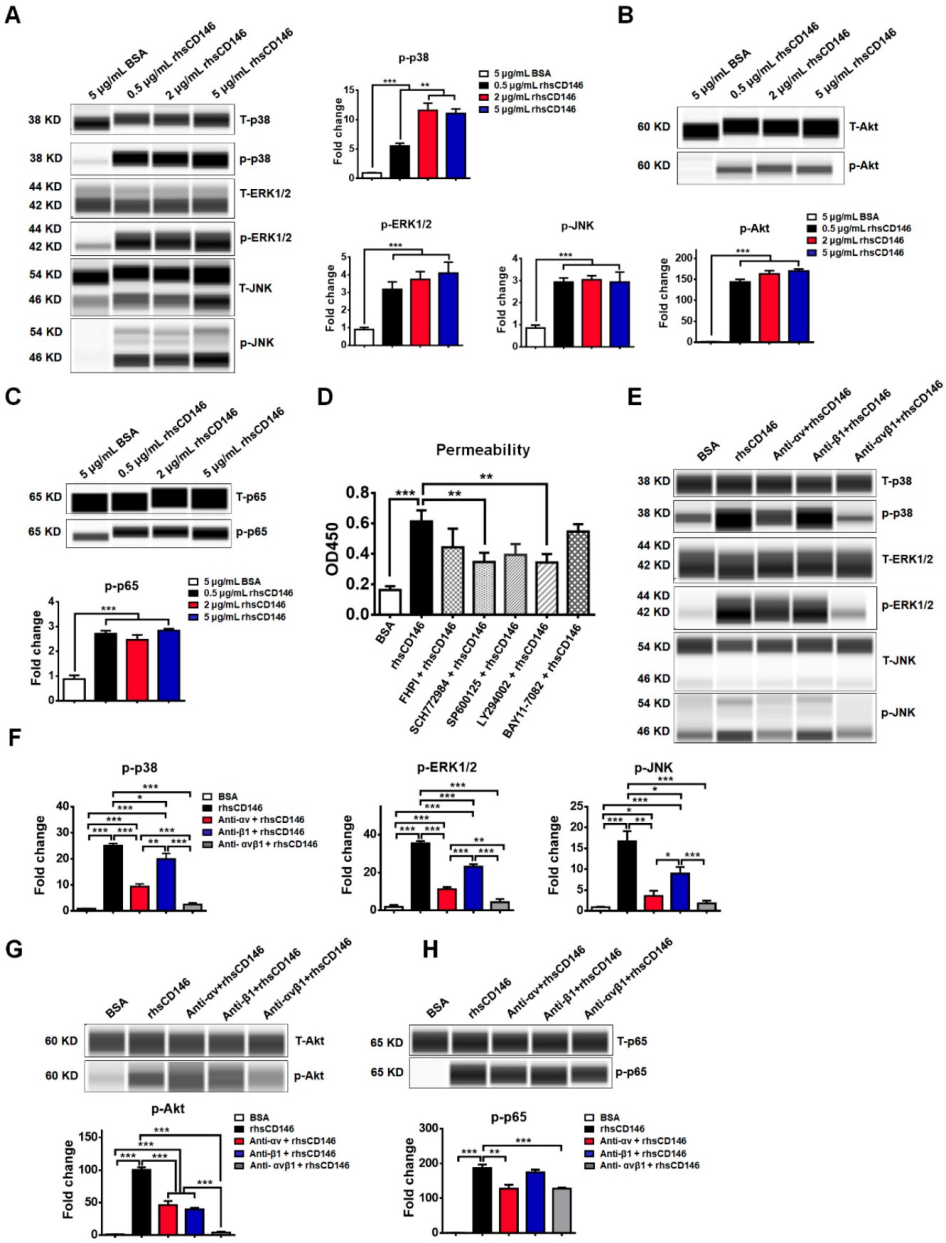
** MAPK, Akt and NF-кB signaling pathways are involved in sCD146-integrin αvβ1 induced hyperpermeability of hCMEC/D_3_ cells. (A-C)** Phosphorylation of p38, ERK1/2, JNK, Akt and NF-кB was induced by treatment with 0.5, 2 or 5 μg/mL rhsCD146 for 10 min in hCMEC/D_3_ cells. At least three independent assays were performed. **(D)** MAPK, Akt and NF-кB signaling pathways are involved in sCD146-induced hyperpermeability of hCMEC/D_3_ cells. hCMEC/D_3_ cells were preincubated with signaling inhibitors 45 min before treatment with 5 μg/mL rhsCD146. The working concentration of signaling inhibitor of p38 (FHPI), JNK (SP600125), and NF-кB (BAY11-7082) is 10 μM, of ERK1/2 (SCH772984) is 2 μM and of Akt (LY294002) is 5 μM. (E-H) rhsCD146-induced phosphorylation of p38, ERK1/2, JNK, Akt and NF-кB was inhibited by anti-integrin αv and β1 antibodies. hCMEC/D_3_ cells were preincubated with 3 μg/mL IgG, anti-integrin αv, anti-integrinβ1 or anti-integrin αvβ1 antibodies for 30 min, and then, 5 μg/mL BSA or rhsCD146 was added to the culture medium and incubated for another 10 min. The cell lysates were harvested for western blot analysis.

MAPK family members ERK1/2 38, JNK 39, p38 40; and Akt 41, NF-кB 42 have been reported to be activated during hyperpermeability or apoptosis of vascular ECs, while inhibition of these signaling pathways significantly blocks these effects. In an* in vitro* study, we found that treatment with rhsCD146 was sufficient to activate these signaling pathways in hCMEC/D_3_ cells (Figure 5A-C and Figure S6). To further evaluate the influence of these signaling pathways for the permeability of hCMEC/D_3_ cells, we inhibited these signaling pathways with related inhibitors. As shown in Figure S7A, the inhibitors significantly decreased rhsCD146-induced abnormal phosphorylation of MAPK, Akt and NF-кB. In *in vitro* permeability assay, we found that rhsCD146-induced hyperpermeability of hCMEC/D3 cells was partially recovered when the phosphorylation of MAPK, Akt and NF-кB was inhibited, especially ERK1/2 and Akt pathways (Figure 5D), and this result was confirmed by TEER analysis (Figure S7B).

We next investigated whether rhsCD146 induced MAPK, Akt and NF-кB signaling pathways activation via integrin αvβ1. As shown in Figure 5D-G and Figure S8, inhibition of αv or β1 significantly reduced the phosphorylation of MAPK and Akt compared with the control. However, the NF-кB signaling pathway seemed to be unaffected or weakly affected by the anti-αv or anti-β1 antibodies, indicating the presence of other regulatory mechanisms.

### Blocking integrin αvβ1 attenuates sCD146-induced BBB permeability

**Figure 6:**
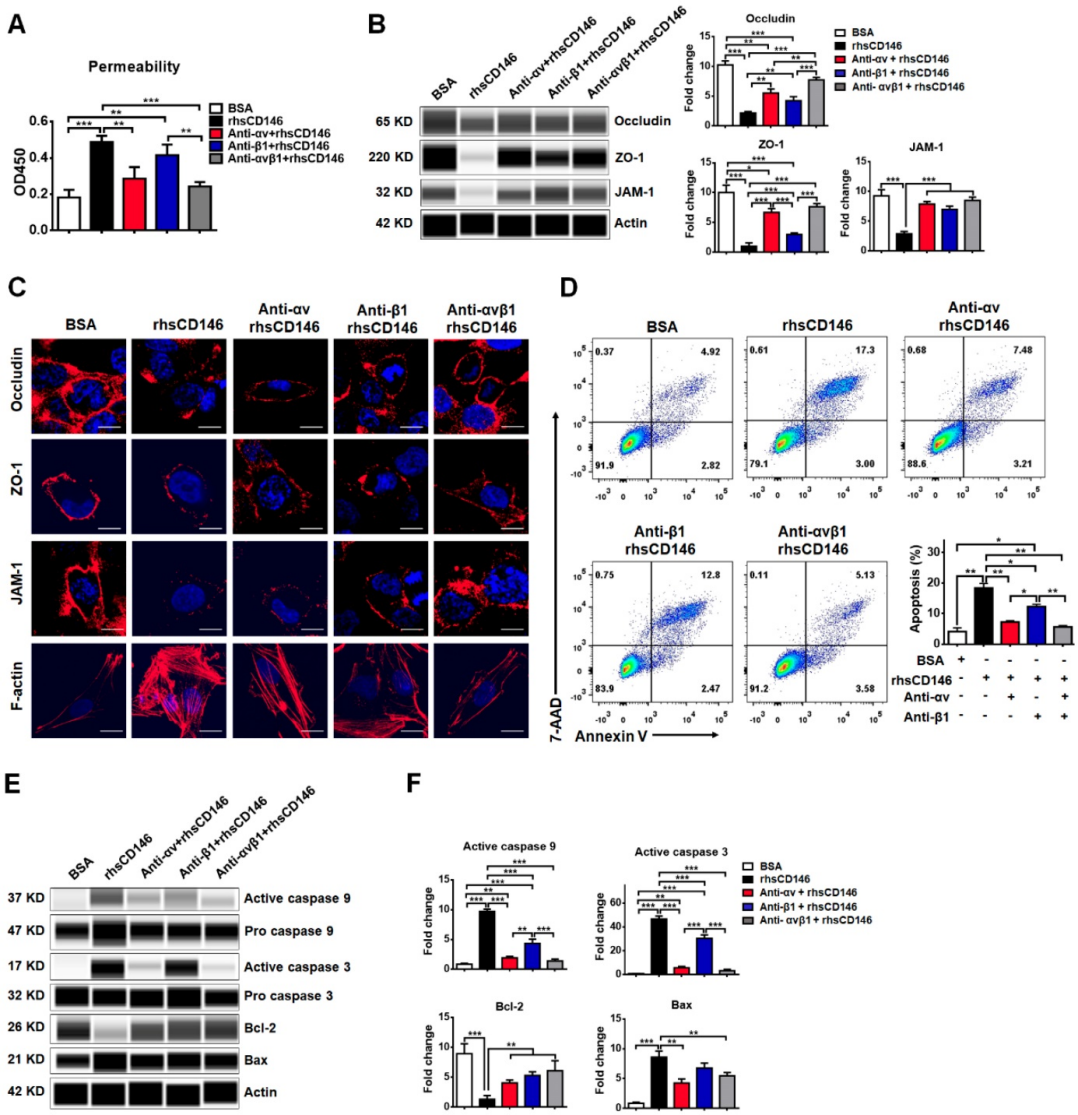
** Blocking integrin αvβ1 attenuates sCD146-induced BBB dysfunction. (A)** rhsCD146-induced paracellular permeability of hCMEC/D_3_ cells was blocked by anti-integrin αv and β1 antibodies. hCMEC/D_3_ cells were seeded to the upper chambers of a transwell system; preincubated with 3 μg/mL IgG, anti-integrin αv or β1 antibody for 30 min; and then cultured with 5 μg/mL BSA or rhsCD146 for another 2 h. Permeability was measured using 0.5 μg/mL HRP. *p<0.05; **p<0.01; and ***p<0.001. **(B)** hCMEC/D_3_ cells were preincubated with 3 μg/mL IgG, anti-integrin αv or β1 antibody for 30 min and then treated with 5 μg/mL BSA or rhsCD146. TJP expression was verified by western blotting. **(C)** Immunofluorescence staining of the TJPs and F-actin in hCMEC/D_3_ cells pretreated with 3 μg/mL IgG, anti-integrin αv or β1 antibody for 30 min and 5 μg/mL BSA or rhsCD146 for 4 h. Bar, 10 μm. **(D)** hCMEC/D_3_ cells were preincubated with 3 μg/mL IgG, anti-integrin αv or β1 antibody for 30 min and treated with 5 μg/mL BSA or rhsCD146 for another 12 h; apoptosis was detected by flow cytometry with Annexin V and 7-AAD. **(E-F)** hCMEC/D_3_ cells were preincubated with 3 μg/mL IgG, anti-integrin αv or β1 antibody for 30 min and treated with 5 μg/mL BSA or rhsCD146 for another 12 h; western blotting was performed to detect the expression of caspase 9, caspase 3, Bcl-2 and Bax.

We have shown that integrin αvβ1 is a major receptor for sCD146 on the membranes of hCMEC/D_3_ cells. We investigated whether sCD146 induces BBB dysfunction via integrin αvβ1. As shown in Figure 6A, rhsCD146 induced the hyperpermeability of hCMEC/D_3_ cells, while this effect was blocked by inhibiting integrin αv, β1, or αvβ1 with antibodies. Western blot and immunocytochemistry analyses showed that treatment with either the anti-integrin αv or β1 antibodies partially blocked the rhsCD146-induced reduction in the expression of TJPs and the reorganization of the actin cytoskeleton to form stress fibers, while using both anti-integrin αv and β1 antibodies almost completely inhibited rhsCD146 function (Figure 6B-C and Figure S9A). In addition, we determined whether preincubation with the anti-integrin αv and β1 antibodies could inhibit the rhsCD146-induced apoptosis of hCMEC/D_3_ cells. Cytometric analysis showed that treatment with anti-integrin αv and β1 antibodies reduced the number of apoptotic cells (Figure 6D). Importantly, blocking integrin αv and β1 significantly reduced the activation of caspase 9 and caspase 3 and the expression of the pro-apoptosis protein Bax, and recovered the expression of the anti-apoptosis protein Bcl-2 (Figure 6E-F and Figure S9B)**.** In conclusion, blocking integrin αvβ1 attenuates sCD146-induced BBB permeability.

## Discussion

An accurate evaluation of BBB integrity is essential for the diagnosis and treatment of neuroinflammatory diseases. However, the identification of new biomarkers with high sensitivity and specificity for BBB disruption is a great challenge. In this study, we showed that CSF sCD146 levels were increased in various active neuroinflammatory diseases based on a larger cohort of patients. Moreover, CSF sCD146 was reduced in remitting MS, indicating the resolution of inflammation. Compared with the routinely utilized parameters in clinical practice, CSF sCD146 showed a high sensitivity (100%) in monitoring BBB damage. An *in vitro* analysis showed that sCD146 actively participated in BBB dysfunction by increasing the permeability and apoptosis of BBB-ECs. Mechanistically, sCD146 was associated with integrin αvβ1, which has been reported to be involved in BBB dysfunction 43, 44. Inhibiting the integrin αv or β1 subunits blocked sCD146-induced BBB dysfunction. Together, our data demonstrate that elevated sCD146 promotes BBB damage and is sensitive for monitoring disease activity.

BBB integrity maintains the homeostasis of the CNS microenvironment, and the disruption of BBB is a hallmark of many neuroinflammatory diseases. Monitoring BBB integrity facilitates predicative disease prognosis and guides treatment. Gd-enhancing MRI is the standard for evaluating BBB disruption 45, 46. Various degrees of BBB lesions can be detected by Gd-enhancing MRI scan. However, in CNS inflammation, even if the BBB lesions are restored, the inflammation will persist for a period. The results of Gd-enhancing MRI reflect morphological changes in BBB structure, and this morphological change generally occurs later than changes in the molecular levels of biomarkers in BBB damage. Therefore, the Gd-enhancing MRI cannot measure the inflammation per se. Other molecules, such as sICAMs, sVCAMs, some cytokines and MMPs, have been studied, and their levels show a positive association with BBB permeability. Although the concentrations of these molecules are increased in the majority of patients, none of these molecules are sufficiently specific for predicting BBB damage accurately 47. In current clinical laboratory examinations, the Q_Alb_ is used to evaluate BBB permeability 48. However, Q_Alb_ has a very low sensitivity in the diagnosis of BBB dysfunction 49, 50. CSF albumin levels could be affected by various factors, such as proteolytic cleavage and uptake by brain macrophages and neurons. In addition, the Q_Alb_ cannot be used to evaluate small local leaks or more widespread areas of a leak or smaller solutes 51. In some cases, although transient destruction of the BBB is repaired subsequently, the resulting pathological damage and inflammation in the CNS can hardly be detected with the Q_Alb_ 52. Therefore, the Q_Alb_ is insufficient for evaluating BBB damage. The development of new biomarkers that are sensitive to BBB leakage and dysfunction is urgently needed. In this study, we provide a promising marker for evaluating BBB damage. By enrolling 823 patients with or without neuroinflammation, we found that sCD146 was markedly elevated in the CSF but not in the serum, suggesting that sCD146 reflected local inflammation, which was confirmed by examining the relationships between sCD146 and CSF inflammatory factors. Moreover, we found that the CSF sCD146 level was positively correlated with the BBB permeability tested by the routinely utilized parameters, such as the Q_Alb_, the IgG index and CSF-MBP. Furthermore, compared to other soluble adhesion molecules that have been reported in the CSF, such as sICAM and sVCAM, CSF sCD146 showed high sensitivity and specificity, a high PPV and a high NPV for measuring BBB damage, indicating that CSF sCD146 is sensitive for monitoring early BBB damage and CNS inflammation. This study provides evidence that sCD146 is a promising marker for measuring BBB damage as well as a potential marker for monitoring inflammation development.

Serum sCD146 is generally known to be a marker that reflects the severity of cardiovascular diseases and tumor development 30, 53, 54. An abnormal elevation in serum sCD146 has been reported to be correlated with the activity of ECs. In early diabetes and diabetic nephropathy, serum sCD146 is significantly increased and positively correlated with the pathological process of disease, suggesting vascular system dysfunction 20, 55. In addition, serum sCD146 is also abnormally elevated in acutely decompensated heart failure, acute coronary syndrome and other cardiovascular diseases, indicating damage to ECs 53, 56. The BBB is a physical and functional vascular structure that is organized by brain ECs that are in contact with various CNS cell types, such as the pericytes and astrocytes. Our previous study showed that membrane-bound CD146 is upregulated in BBB-ECs during CNS inflammation and mediates the exacerbation of inflammation. However, whether sCD146 is involved in BBB dysfunction is still unknown. In our previous study, we showed that sCD146 promotes CNS inflammation by facilitating the transmigration of leukocytes across BBB-ECs 18. Interestingly, in the present study, we showed that sCD146 directly promotes BBB hyperpermeability, as supported by several lines of evidence. First, sCD146 inhibits the expression of TJPs in BBB-ECs and induces the reorganization of the actin cytoskeleton to form stress fibers. Second, sCD146 promotes the apoptosis of BBB-ECs in a dose-dependent manner. Third, sCD146 activates several BBB-ECs signaling pathways, which have been reported to be involved in ECs activation. Finally, sCD146 is associated with integrin αvβ1, suggesting the active and important role of sCD146 in BBB dysfunction.

Because the BBB is an interface between the brain parenchyma and the peripheral circulation, maintaining BBB integrity is pivotal for CNS homeostasis. Malfunctions in any component of the BBB, such as brain microvascular ECs, astrocytes, pericytes and the basal membrane, could lead to critical changes in BBB function. BBB-ECs are the primary component of the BBB. The special structural features of BBB-ECs, such as their low rate of pinocytosis, higher mitochondrial content and lack of fenestrations, distinguish BBB-ECs from peripheral vascular ECs. Under physiological conditions, BBB-ECs express lower levels of adhesion molecules, which limits the entry of immune cells into the CNS. However, BBB-ECs are susceptible to damage under pathological settings, such as viral infection, trauma or systemic inflammation. The upregulation of adhesion molecules, loss tight junctions, or redistribution of cytoskeletal proteins can impair the integrity and/or permeability of the BBB. Pericytes are also important compounds for BBB development and function. Pericytes contact BBB-ECs to downregulate BBB permeability by releasing PDGF-Rβ and TGF-β. In our recent study, we found that the adhesion molecule CD146 is important for the development and maturation of BBB. The overexpression of CD146 on developing BBB-ECs triggers the recruitment of pericytes to form tight endothelial-pericyte contacts. In the mature stage, CD146 is downregulated on BBB-ECs while remaining highly expressed on pericytes. During conditions of inflammation, CD146 expression is upregulated on BBB-ECs and subsequently promotes the transmigration of inflammatory cells across the BBB 16. Moreover, we found that the soluble form of CD146 also promotes inflammation by facilitating leukocyte infiltration 18. Previous studies reported that sCD146 displays angiogenic properties by associated with angiomotin and promotes neovascularization in experimental hind-limb ischemia 57, 58. In this study, we found that sCD146 induced the permeability of BBB by disrupting BBB TJPs and promoting BBB-ECs apoptosis. sCD146 interacts with integrin αvβ1 and promotes the activation of the MAPK, Akt and NF-кB signaling pathways, which are associated with ECs activation 59-61. These data suggest that sCD146 has multiple functions during BBB dysfunction. Therefore, regardless of whether CD146 is membrane-bound or soluble, CD146 is actively involved in BBB dysfunction, and targeting CD146 would be a promising strategy for dampening neuroinflammation.

Overexpressed CD146 sheds in settings in which MMPs are present, and the soluble form of CD146 then distributes into the body fluid 62. In neuroinflammatory diseases, we found no significant increase in sCD146 in the serum, suggesting that the level of sCD146 that originates from shedding might be far lower than that of the sCD146 pool in the serum or that sCD146 diffuses into other body fluids, such as the CSF. In normal human CSF, very low levels of sCD146 can be detected. During the development of the BBB damage and neuroinflammation, CSF sCD146 is significantly increased. However, the origin of CSF sCD146 in these patients is still unknown. In this study, we found that sCD146 binds to BBB-ECs and then translocates to the cytoplasm, suggesting receptor-mediated transmembrane transport of sCD146. The second origin of CSF sCD146 may be the ECs of the choroid plexus. CSF is produced in the choroid plexus, which comprises the pia mater, ECs and the ependymal epithelium. Our unpublished data indicate that the ECs and ependymal epithelium of the choroid plexus express high levels of membrane CD146 in experimental autoimmune encephalomyelitis mice compared to normal mice. This expression would be one possible origin of CSF sCD146 during inflammation. Thirdly, during the development of neuroinflammation, brain tissue appears obvious lesions that form a local inflammatory response, which could damage the external side of brain blood vascular endothelial cells, resulting in the shedding of sCD146. Further studies are needed on the origin of CSF sCD146.

In summary, this study provides convincing clinical evidence to show that CSF sCD146 is sensitive for monitoring BBB damage and neuroinflammation. Furthermore, we demonstrate that sCD146 directly compromises the barrier function of human brain ECs via integrin αvβ1-mediated intracellular signaling events. Based on these findings, we suggest CSF sCD146 as a promising diagnostic index for evaluating early BBB damage in multiple neurological diseases and as a potential therapeutic target for various neuroinflammatory diseases.

## Methods

### Patients and samples

This study enrolled 823 patients from 30 provinces of China who were diagnosed with or without neuroinflammatory diseases between 2011 and 2017. Their clinical diagnosis was confirmed at Peking University First Hospital. Written informed consent was obtained, and ethical approval was granted by the Ethics Committee of the Peking University First Hospital and Institute of Biophysics, Chinese Academy of Sciences before human sample collection. We also obtained written informed consent from guardians on behalf of the children who participated in this study. CSF samples were collected by lumbar puncture for diagnostic or surgical purposes. CSF samples were immediately centrifuged at 4 °C at 4000 rpm for 5 min. For serum collection, blood samples were taken from an antecubital vein and centrifuged at 4 °C at 4000 rpm for 5 min. The supernatants of the CSF and serum samples were separated as soon as possible, coded, frozen, stored at -80 °C and thawed just before analysis to avoid loss of biological activity.

According to etiology, the patients were divided into four groups: IIDD, CNSI, PNS and NIND. IIDD included relapsing MS and neuromyelitis optical spectrum disorders (NMOSD). Relapsing MS (n=93) and remitting MS (n=44) were diagnosed according to the McDonald criteria 63. Relapsing MS was defined as obvious neurological impairment or the appearance of a new symptom or abnormality attributable to MS, with symptoms lasting 24 h and preceded by stability for at least 1 month. All the enrolled relapsing MS patients had disease progression ranging from 1 to 12 years. These patients who were in relapsing stages had received treatments with anti-inflammation reagents such as glucocorticoids, fingolimod, azathioprine, cyclophosphamide or amethopterin. Patients with remitting MS received anti-inflammation treatments before lumbar puncture. CSF and serum samples were collected from remitting MS patients within 2 weeks of the onset of acute or subacute exacerbation and from remitting MS patients who were stable for at least 2 weeks. The assessment of NMOSD (n=43) followed the diagnostic criteria by Wingerchuk in 2015 64. NMOSD progression ranged from 1 to 5 years. Patients received treatments with glucocorticoids, intravenous immunoglobulin and plasma exchange. CSF and serum samples were collected from NMOSD patients within 2 weeks of the onset of acute exacerbation. CNSI included intracranial mycoplasma infection (n=45), viral encephalitis (n=67), virus- or bacteria-induced myelitis (n=31) and tuberculous meningitis (n=67). Disease progression ranged from 1 to 13 months. These patients with CNSI had received anti-pathogen therapy, a low dose of glucocorticoids or other immunosuppressive agents. CSF and serum samples were collected within 2 weeks of onset. PNS included chronic inflammatory demyelinating polyneuropathy (n=71), acute inflammatory demyelinative polyradiculoneuropathy (n=60), myasthenia syndrome (n=45) and amyotrophia (n=40). PNS (n=216) progression ranged from 1 to 16 months. Patients had received treatments with glucocorticoids, intravenous immunoglobulin, plasma exchange, cyclosporine, cyclophosphamide, tacrolimus, azathioprine or methotrexate. CSF and serum samples were collected from PNS patients within 2 weeks of onset. In this study, all patients with neuroinflammatory diseases presented with obvious BBB damage via Gd-MRI. Patients with NIND served as controls without any CNS demyelinating lesions in their MRI scans. NIND included varicose veins of the lower extremities (n=46), osteoarthropathy (n=49), uterine prolapse (n=62) and inguinal hernia (n=60). Twenty-three patients experienced hypertension or diabetes. Patients with NIND had received antibiotic therapy. CSF samples were obtained from surgical spinal anesthesia. The details pertaining to other information regarding the participants are shown in Table 1.

### Detection of sCD146 by ELISA

The concentration of sCD146 in CSF or serum was measured with a sandwich ELISA protocol that was modified from that of a previous report 18. Briefly, anti-CD146 mAb AA1 (2 μg/mL) was coated onto the ELISA plate as a capture antibody, and HRP-conjugated AA98 (1 μg/mL) served as the detection antibody. Recombinant human sCD146 (rhsCD146) was used to generate a standard curve from 80 to 2.5 ng/mL. CSF samples were diluted 1:4, and serum samples were diluted 1:20 in a protein stabilizer (Biopanda, UK). A ready-to-use solution of 3,30,5,50-tetramethylbenzidine (TMB) was used as a substrate for the HRP enzyme, and 2 mol/L H_2_SO_4_ was used to stop the reaction. The OD value was measured at a wavelength of 450 nm.

### CBA immunoassay for inflammation-related factors

CSF supernatants were diluted 1:1 with PBS and analyzed simultaneously for 9 different inflammation-related factors, namely, MMPs (MMP2 and MMP9), sICAM, sVCAM, IL-1β, IL-17A, IFN-γ, IL-10 and TNF-α. The multifactor detection kit (Biolegend, #740001, USA) was used according to the manufacturer's instructions. All the data from the samples were analyzed using FlowCytomixPro software.

### Immunocytochemistry

The human BBB endothelial cell line hCMEC/D_3_ was kindly provided by Prof. Pierre-Olivier Couraud (Universite´ Rene´ Descartes, Paris, France). MycoAlert (Lonza, LT07-118, Switzerland) was used to verify a lack of mycoplasma contamination every 2 months. hCMEC/D_3_ cells were grown on slides in 24-well plates and stimulated with the appropriate conditional medium. Treated cells were fixed with 4% paraformaldehyde (PFA) for 20 min, permeabilized with 0.1% Triton X-100 for 3 min and then washed with PBS. Cells were blocked with 5% skim milk in PBS for 30 min at 37 °C. Cells were incubated overnight at 4 °C with primary antibodies to occludin (Abcam, #ab31721, UK), JAM-1 (Abcam, #ab180821, UK) and ZO-1 (Invitrogen, #14-9776-80, USA). Cells were incubated with appropriate fluorescence-labeled secondary antibodies for 45 min at 37 °C. Nuclei were counterstained with DAPI. Cells were mounted with cover slides in antifading agent (Merck, #10981, Germany). In addition, cytoskeletal protein F-actin was directly stained with phalloidin (Biolegend, #424203, USA) for 30 min at 37 °C. Cells were analyzed using a laser scanning confocal microscope (OLYMPUS FV1000).

### *In vitro* BBB permeability analysis

An *in vitro* BBB permeability assay was performed using a transwell system (3-μm pore filters, Corning Costar, USA). hCMEC/D_3_ cells were seeded into the upper chamber, and confluent cells were treated with appropriate conditional medium, followed by the addition of HRP in to the upper chamber. One hundred microliters of culture medium were collected from the bottom chamber, and TMB solution was used to measure the OD value (450 nm) at the appropriate time point.

### The TEER value measurement

The TEER value was measured with cell resistance meter (KINGTECH, #RE1600, China) according to the manufacturer's instructions. The TEER _EC_ = (TEER _EC + filter_ - TEER _filter_) × S (membrane area), unit: Ω**·**cm^2^.

### *In vitro* apoptosis assay

To assess hCMEC/D_3_ apoptosis, the Annexin V-FITC and 7-AAD apoptosis kit (Sungenebiotech, #AO2001-02A-H, China) was used for flow cytometric detection. Briefly, cells were washed with cold PBS and suspended in 1× binding buffer. A total of 1×10^6^ cells in 100 μL were used for analysis. A total of 5 μL of Annexin V-FITC was added to the cells for 10 min at room temperature, and the mixture was protected from light. After incubation, 5 μL of 7-AAD solution was added for another 5 min at room temperature. Without washing the cells, PBS was added to the cells up to a volume of 500 μL, and the cells were analyzed by flow cytometry within 1 h.

### Coimmunoprecipitation

For the coimmunoprecipitation assay, the cells were lysed with lysis buffer containing 20 mmol/L Tris-HCl (pH 7.4), 150 mmol/L NaCl, 1 mmol/L CaCl_2_, 2 mmol/L MnCl_2_, 1.5% Triton X-100, 2 mmol/L PMSF, and 10 μg/mL leupeptin, and the lysates were precleared with various types of nonspecific IgG antibodies and Protein G-plus agarose beads. The precleared cell lysates, each containing 1 mg of total protein, were separately incubated for 4 h at 4 °C with human Fc or Fc-sCD146, followed by the addition of 25 μL of precleared Protein G-plus agarose beads and incubation overnight at 4 °C. The proteins eluted from the Protein G-plus agarose beads were subjected to SDS-PAGE under reducing conditions and detected with an anti-integrin αv (Abcam, #ab16821, UK), β1 (Abcam, #ab24693, UK) or anti-Fc antibody (Abcam, #ab109489, UK).

### Cell adhesion assay

A 96-well plate was precoated with 10 μg/mL rhsCD146. hCMEC/D_3_ cells were harvested, preincubated in serum-free medium with various treatments for 30 min on ice, seeded into the plates, and incubated for another 30 min at 37 °C. Nonattached cells were removed by vigorous agitation and aspiration. Attached cells were counted under an optical microscope.

### Automated western blot

Cells were treated under various conditions and then lysed with a cell lysis reagent (SIGMA, #018M4125V, USA). The simple western immunoblots were performed on a Wes (ProteinSimple) using the Size Separation Master Kit with Split Buffer (12-230 KD) according to the manufacturer's standard instruction. The antibodies used included the following: p38 (CST, #9218, USA); phospho-p38 (CST, #4511, USA); ERK1/2 (CST, #4695, USA); phospho-ERK1/2 (CST, #9101, USA); JNK (CST, #9252S, USA); phospho-JNK (CST, #4668S, USA); Akt (CST, #2920S, USA); phospho-Akt (CST, #4060, USA); p65 (CST, #6956S, USA); phosphor-p65 (CST, #3033S, USA); β-actin (ABGENT, #AM1021B, USA); and anti-angiomotin (Abcam, #ab85143, UK).

### Statistical analysis

The results are expressed as the mean±SD. The nonparametric Mann-Whitney U test was employed for comparisons of the cytokine levels in each group. Correlations between the sCD146 level in the CSF and clinical indexes were evaluated using multiple linear regression and Spearman's Rank correlation coefficient. The cut-off value of sCD146 was calculated as the mean±2SD of the NIND group. We assessed the sensitivity, PPVs and NPVs of each biomarker to distinguish neuroinflammatory diseases from NIND. SPSS 11.0 for Windows was used to perform the analysis. The criterion for statistical significance was defined as p<0.05.

## Supplementary Material

Supplementary figures and tables.

## Figures and Tables

**Table 1:** Clinical information of patients with different types of non- or neuroinflammatory diseases

	NIND	IIDD	CNSI	PNS	Remitting MS
No. of patients	217	136	210	216	44
Sex (F/M)	103/114	75/61	101/109	84/132	30/14
Age (years)	47.4±17.3	38.7±15.6	39.2±18.5	46.8±17.2	37.8±15.0
NO. of OCB	40	95	109	60	2
Q_Alb_ (×10^-3^)	4.6±5.2	5.9±3.3	10.5±10.2	8.7±7.3	6.3±5.2
IgG synthesis (mg/24 h)	4.2±4.7	8.1±10.2	25.4±28.8	38.9±46.9	10.2±13.2
IgG index	0.86±0.38	0.87±0.32	1.05±0.24	1.18±0.90	1.06±0.54
C-MBP (μg/L)	4.5±3.5	4.8±3.7	7.4±4.9	7.7±4.2	5.8±3.6
S-MBP (μg/L)	6.0±4.3	7.4±4.2	6.6±4.0	8.4±3.7	5.8±3.5
C-MBP/S-MBP	1.1±1.2	0.7±0.5	1.6±1.8	1.0±0.5	1.2±1.2

Abbreviations: NIND, non-inflammatory neurological diseases; IIDD, idiopathic inflammatory demyelinating diseases; CNSI, central nervous system infection; PNS, peripheral neuropathies; MS, multiple sclerosis; OCB, oligoclonal bands; Q_Alb_, albumin quotient; C-MBP, myelin basic protein in CSF; S-MBP, myelin basic protein in serum; CSF, cerebrospinal fluid.

**Table 2:** Correlation analysis between CSF sCD146 and various clinical parameters in neuroinflammatory diseases

Disease	CSF sCD146	Correlation coefficient	P value	Number
NIND	Age	0.12	0.42	217
	Q_Alb_	0.09	0.47	
	IgG index	-0.18	0.22	
	IgG synthesis	-0.02	0.90	
	C-MBP	-0.11	0.43	
	S-MBP	-0.12	0.37	
	C-MBP/S-MBP	0.32	0.19	
	Sera sCD146	0.19	0.16	
IIDD	Age	0.16	0.07	136
	Q_Alb_	0.48	***<0.001	
	IgG index	0.20	*0.02	
	IgG synthesis	0.42	***<0.001	
	C-MBP	0.30	***<0.001	
	S-MBP	-0.05	0.60	
	C-MBP/S-MBP	0.01	0.94	
	Sera sCD146	0.07	0.54	
CNSI	Age	0.12	0.48	210
	Q_Alb_	0.37	*0.02	
	IgG index	-0.03	0.74	
	IgG synthesis	0.36	*0.01	
	C-MBP	0.33	*0.02	
	S-MBP	-0.02	0.59	
	C-MBP/S-MBP	0.10	0.52	
	Sera sCD146	0.14	0.23	
PNS	Age	0.06	0.67	216
	Q_Alb_	0.31	*0.03	
	IgG index	0.004	0.98	
	IgG synthesis	0.39	**0.004	
	C-MBP	0.43	**0.002	
	S-MBP	0.11	0.43	
	C-MBP/S-MBP	0.16	0.25	
	Sera sCD146	0.03	0.29	

**Table 3:** Correlation between sCD146 and neuroinflammation-related factors in CSF from patients with neuroinflammatory diseases

Disease	CSF sCD146	Correlation coefficient	P value	Number
Remitting MS	sVCAM (7.5±8.0)	0.12	P>0.05	44
	sICAM (20±1.2)	0.33	P>0.05	
	MMP2 (13.4±14.1)	-0.29	P>0.05	
	MMP9 (0.15±0.40)	-0.17	P>0.05	
	IL-1β (4.22±2.27)	0.24	P>0.05	
	IL-17A (11.5±19.2)	-0.19	P>0.05	
	IFN-γ (5.2±4.1)	0.31	P>0.05	
IIDD	sVCAM (**21.0±25.3)	0.56	***<0.001	93
	sICAM (***259.2±276.3)	0.55	***<0.001	
	MMP2 (***47.1±30.8)	0.49	**0.002	
	MMP9 (***1.06±1.65)	0.58	***<0.001	
	IL-1β (*6.09±3.15)	0.38	*0.02	
	IL-17A (***37.3±27.3)	0.45	**0.005	
	IFN-γ (*11.6±10.9)	0.37	*0.02	
CNSI	sVCAM (***30.3±29.5)	0.61	**0.006	109
	sICAM (**283.1±258.4)	0.50	*0.03	
	MMP2 (**44.1±27.9)	0.53	**0.007	
	MMP9 (**1.29±1.78)	0.59	**0.007	
	IL-1β (***7.37±3.59)	0.49	*0.02	
	IL-17A (***55.9±31.3)	0.50	*0.01	
	IFN-γ (***12.2±8.8)	0.43	*0.04	

The concentration unit of sVCAM, MMP2, MMP9: ng/mL; The concentration unit of sICAM, IL-1β, IL-17A, IFN-γ: pg/mL; Values are expressed as the mean ± SD. *p<0.05; **p<0.01; and ***p<0.001. Data are representative of three independent experiments.

**Table 4:** Sensitivity, specificity, PPV and NPV of CSF sCD146 and other molecules in the diagnosis of BBB damage

		Neuroinflammatory diseases		
	Sensitivity (%)	Specificity (%)	PPV (%)	NPV (%)
CSF sCD146 (<10.5 ng/mL)	100 (562/562)	93.1 (202/217)	97.4 (562/577)	100 (202/202)
Q_Alb_ (<5 × 10^-3^)	46.3 (260/562)	64.1 (139/217)	76.9 (260/338)	31.5 (139/441)
IgG synthesis (<30 mg/24 h)	31.7 (178/562)	86.6 (188/217)	85.9 (178/207)	32.9 (188/572)
IgG index (<0.85)	69.6 (391/562)	41.2 (120/217)	80.2 (391/488)	41.2 (120/291)
CSF-MBP (<3.5 μg/mL)	54.7 (307/562)	64.5 (140/217)	79.9 (307/384)	35.4 (140/395)
Serum MBP (<2.5 μg/mL)	73.9 (415/562)	55.8 (121/217)	81.2 (415/511)	45.1 (121/268)
OCB	47 (264/562)	81.6 (177/217)	86.8 (264/304)	37.3 (177/475)
sVCAM (<23.5 ng/mL)	38.8 (78/202)	100 (44/44)	100 (78/78)	26.2 (44/168)
sICAM (<22.4 pg/mL)	74.4 (150/202)	100 (44/44)	100 (150/150)	45.8 (44/96)
MMP2 (<41.6 ng/mL)	72.2 (146/202)	95.5 (42/44)	98.6 (146/148)	42.9 (42/98)
MMP9 (<0.95 ng/mL)	28.9 (58/202)	95.5 (42/44)	96.7 (58/60)	22.6 (42/186)
IL-1β (<8.76 pg/mL)	13.3 (27/202)	93.2 (41/44)	90.0 (27/30)	19.0 (41/216)
IL-17A (<49.9 pg/mL)	41.1 (83/202)	90.9 (40/44)	95.4 (83/87)	25.2 (40/159)
IFN-γ (<13.4 pg/mL)	33.3 (67/202)	100 (44/44)	100 (67/67)	24.6 (44/179)

Abbreviations: PPV, positive predictive value, calculated from the number of neuroinflammatory patients who test positive relative to the total number of patients who test positive; NPV, negative predictive value, calculated from the number of NIND patients who test negative relative to the total number of patients who test negative. The cut-off values of sCD146, sVCAM, sICAM, MMP2, MMP9, IL-1β, IL-17A and IFN-γ are represented by the mean ± 2SD.

## Abbreviations

BBB: blood-brain barrier
sCD146: soluble CD146
ECs: endothelial cells
CSF: cerebrospinal fluid
MS: multiple sclerosis
NIND: non-inflammatory neurological diseases
CNS: central nervous system
IIDD: idiopathic inflammatory demyelination diseases
CNSI: central nervous system infection
PNS: peripheral neuropathies
OCB: oligoclonal bands
Q_Alb_: albumin quotient
MBP: myelin basic protein
MMPs: matrix metalloproteinases
PPV: positive predictive value
NPV: negative predictive value
TEER: transendothelial electrical resistance
TJPs: tight junction proteins
ZO-1: zonula occludens-1
JAM-1: junctional adhesion molecule-1
BBB-ECs: blood-brain barrier endothelial cells
NMOSD: neuromyelitis optica spectrum disorders
