# Supplementary material for: Soluble CD146, a cerebrospinal fluid marker for neuroinflammation, promotes blood-brain barrier dysfunction
Source: Theranostics. 2020 Jan 1;10(1):231–46. doi: 10.7150/thno.37142 (PMC6929609; doi:10.7150/thno.37142)
Supplement: Supplementary file 1 — Supplementary figures and tables. [file thnov10p0231s1.pdf]

Supplementary information

Soluble CD146, a cerebrospinal fluid marker for neuroinflammation, promotes blood-brain barrier dysfunction

Daji Wang[1]*, Hongxia Duan[2]*, Jing Feng[2], Jianquan Xiang[4], Liqun Feng[5], Dan Liu[2], Xuehui Chen[2], Lin Jing[2], Zheng Liu[2], Dexi Zhang[2], Hongjun Hao[3†], Xiyun Yan[1,2†]

Affiliations:

1. **Savaid Medical School, University of Chinese Academy of Sciences.**
2. **Key Laboratory of Protein and Peptide Pharmaceutical, Institute of Biophysics, Chinese Academy of Sciences.**
3. **Neuroimmunology Laboratory, Peking University First Hospital.**
4. **School of Basic Medical Sciences, Southwest Medical University.**
5. **Beijing Anzhen Hospital, Capital Medical University.**

*** Contributed equally as first authors**

† Contributed equally as last authors

Hongjun Hao, MD, Email: haohj1963@126.com

Xiyun Yan, MD, Email: yanxy@ibp.ac.cn

Table. S1 sCD146 reflects the progression of neuroinflammatory diseases

Diseases	Number	CSF sCD146	Sera sCD146
NIND	217	4.7 ± 2.9	380.5 ± 97.7
Relapsing MS	93	***44.3±18.9	378.1±109.2
Remitting MS	44	4.6±3.5	379.4±110.6

The values are expressed as the mean ± SD. *p<0.05; **p<0.01; and ***p<0.001. The data are representative of three independent experiments.

Table. S2 The primers of integrins for qPCR

Primer name	Forward primer (5′ - 3′)	Reverse primer (5′ - 3′)
Integrin α1	GGAGAACAGAATTGGTTCCTAC	CGGAGCTCCATCACGATCATTA
Integrin α2	GAATCGCGATGTTGGTGAGC	CTGTGTCCACACCTGAGCTT
Integrin α3	CCCATATGCCACTCTCTGCC	GTAGGGCCACTCCAGACCTA
Integrin α4	ATGCTGCAAGATTTGGGGAA	GCACCAACTGCTACATCTAC
Integrin α5	GGCTTCAACTTAGACGCGGA	GGCCGGTAAAACTCCACTGA
Integrin α6	GGAGCAACAGCAAACAGGTG	CCGAATCCCATTGCTTTGGC
Integrin αv	AGGCACCCTCCTTCTGATCC	GCGGGTAGAAGACCAGTCAC
Integrin β1	ACGGACGTAAAGCTGGTCTC	TTGCACGGGCAGTACTCATT
Integrin β3	ACCAGTAACCTGCGGATTGG	TCCGTGACACACTCTGCTTC
Integrin β4	CACAGGTGGCATGGTTGTTG	AAGCTGCTCTCCATGACCAC
Integrin β5	AGCGGCGACACACTAGGA	GAGCACCAGGCACATTTTGG
Integrin β6	TCATAAAGCCTGTGGGGCTG	TGAGAAATCTCCGCAGAGCAG
Integrin β8	AGCTGTTACTGCTGCTCCTG	TCCAAGACGAAAGTCACGGG

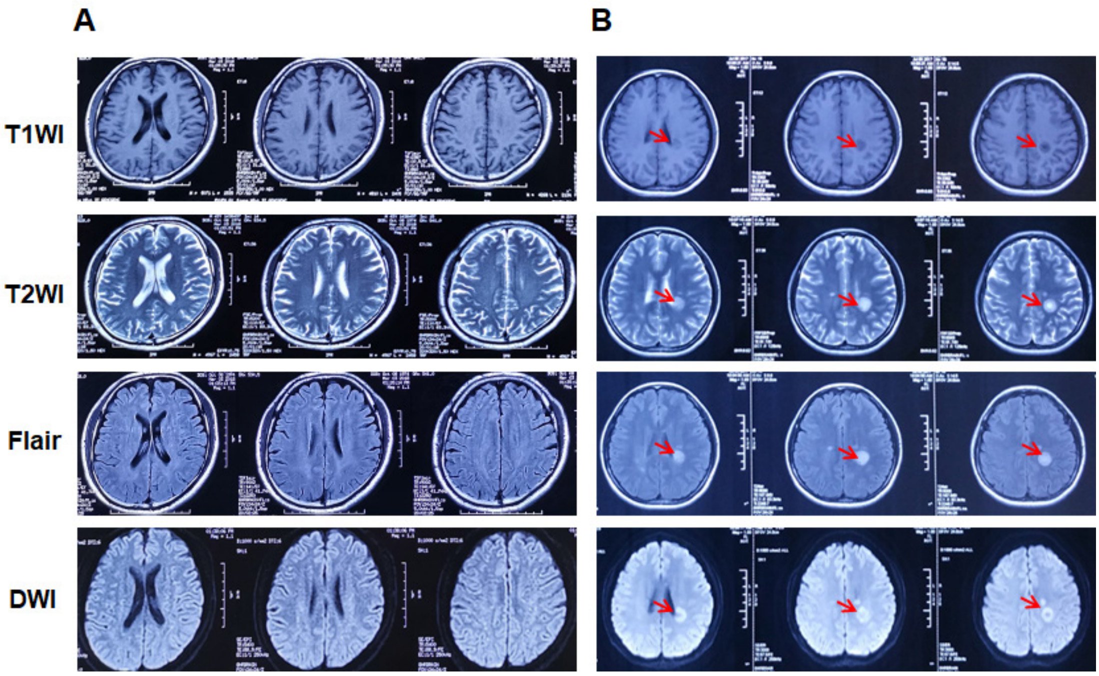

Figure S1. The MRI data of (A) healthy people and (B) patient with active multiple sclerosis.

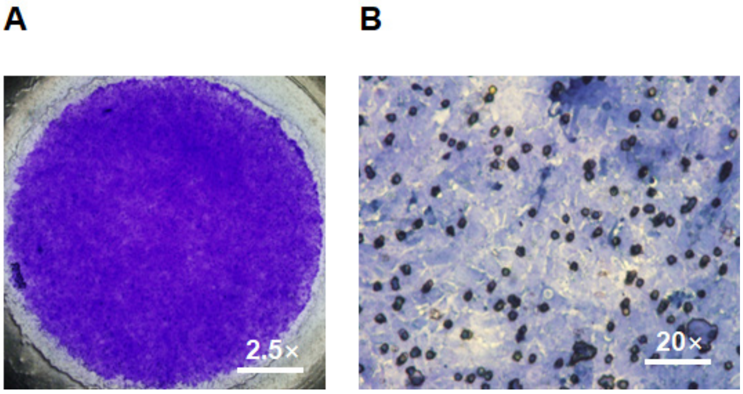

Figure S2. Crystal violet staining for hCMEC/D_3 cells. A total of 1×10^5 cells were seeded into the upper chamber of the transwell system (3 μm), and crystal violet staining was used to confirm 100% cell confluence.

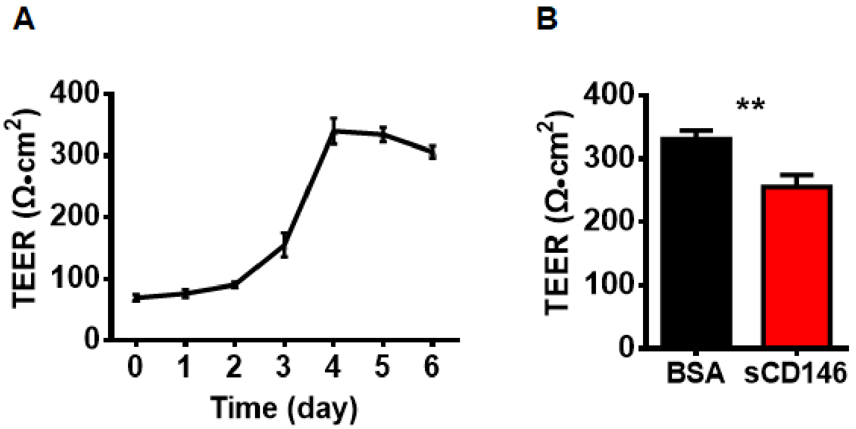

Figure S3. sCD146 promotes BBB permeability *in vitro*. (A) A total of 1×10^5 hCMEC/D_3 cells were seeded into the upper chamber of the transwell system (3 μm), and the TEER value was detected. (B) The TEER value of BBB model was measured after cells were incubated with 5 μg/mL BSA or rhsCD146 for 2 h. *p<0.05; **p<0.01; and ***p<0.001.

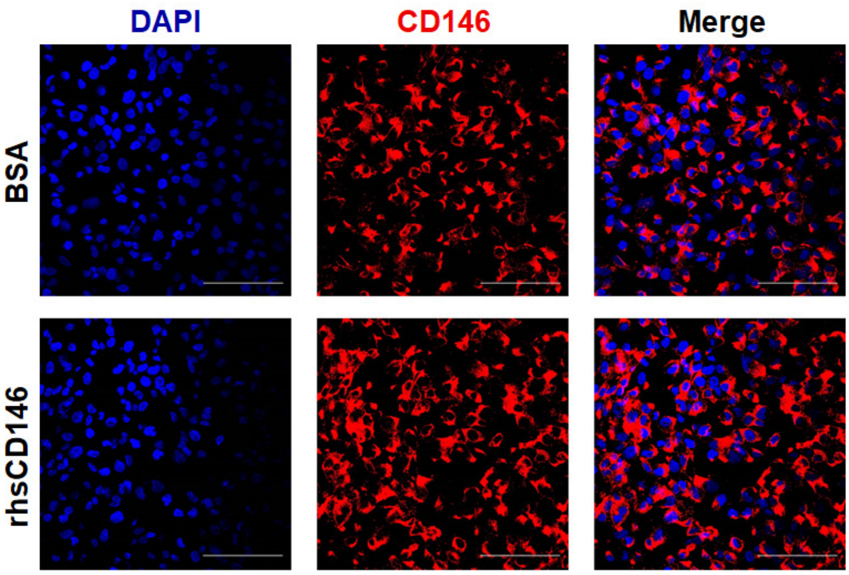

Figure S4. sCD146 binds to unknown receptors on the membranes of hCMEC/D_3 cells. Immunofluorescence staining of sCD146 after hCMEC/D_3 cells were treated with 5 μg/mL BSA or rhsCD146 for 1 h. Bar, 100 μm.

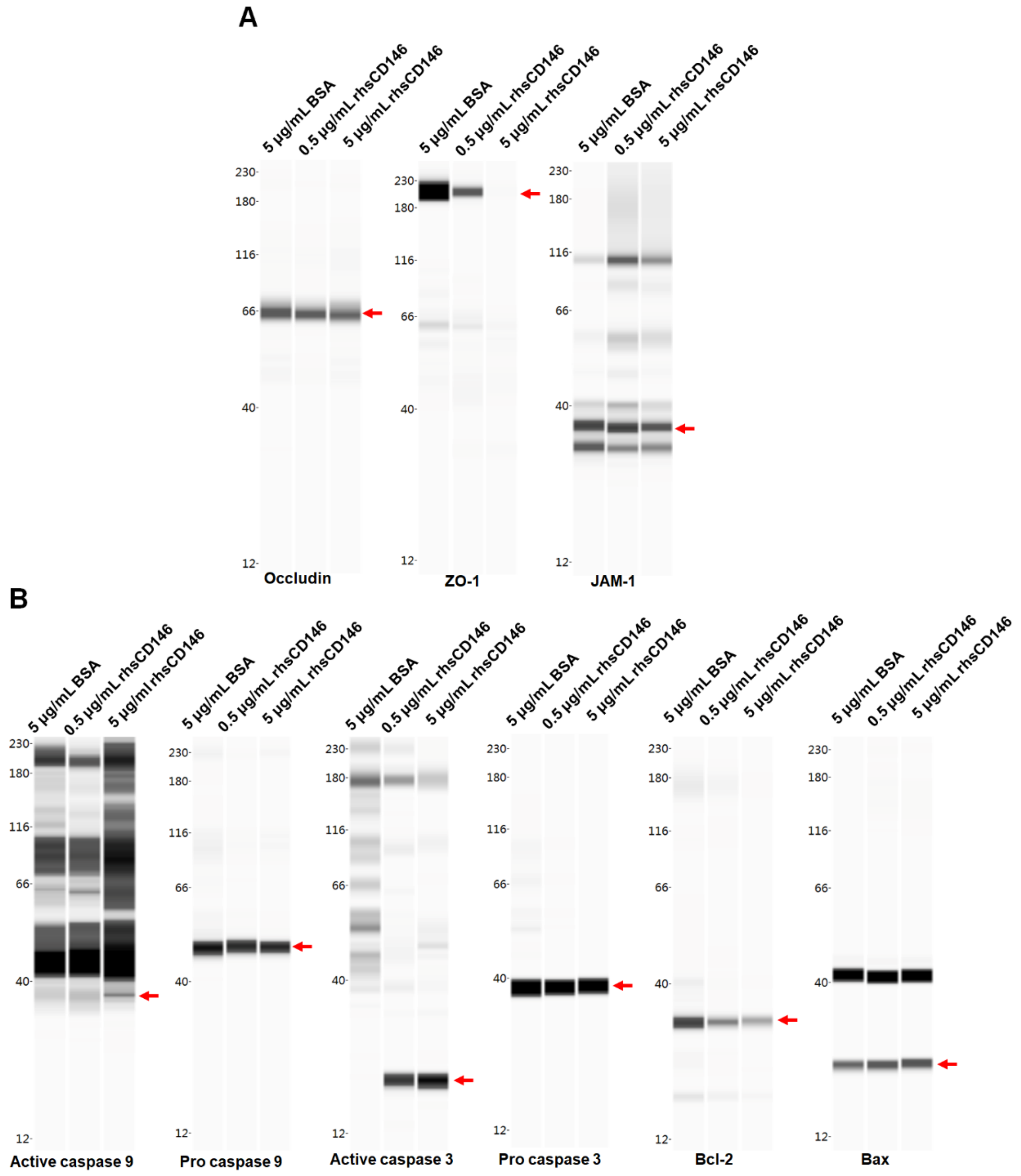

Figure S5. The expression of TJPs and apoptosis-related molecules in hCMEC/D$_3$ cells after treatment with different concentrations of rhsCD146. (A) hCMEC/D$_3$ cells were preincubated with 5 μg/mL BSA, 0.5 μg/mL or 5 μg/mL rhsCD146, and TJP expression levels were verified by western blotting. (B) hCMEC/D$_3$ cells were treated with 5 μg/mL BSA, 0.5 rhsCD146 or 5 μg/mL rhsCD146 for 12 h, and cell lysates were used to detect the expression of caspase 9, caspase 3, Bcl-2 and Bax.

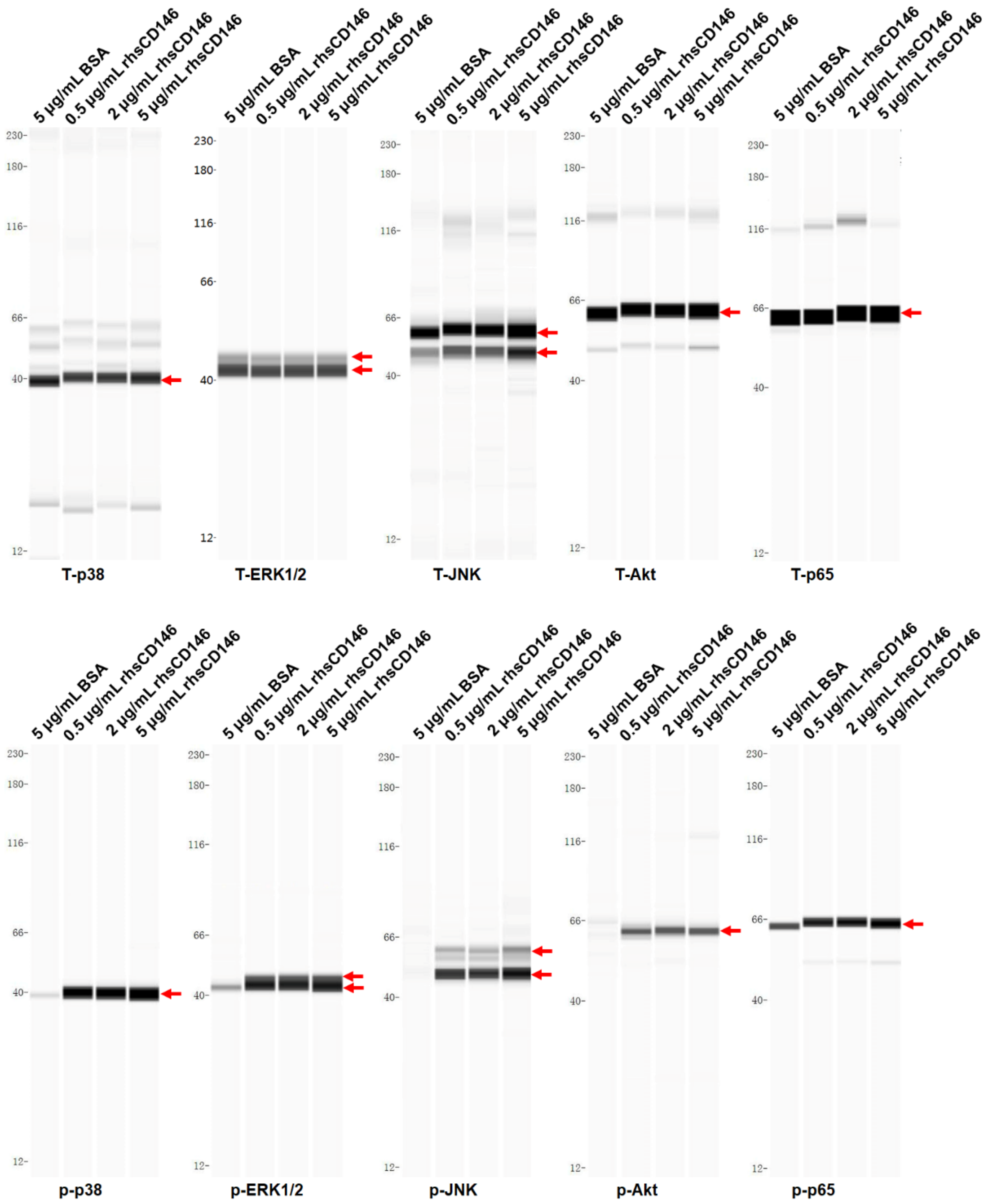

Figure S6. The phosphorylation of p38, ERK1/2, JNK, Akt and NF-κB was induced by treatment with 0.5, 2 or 5 μg/mL rhsCD146 for 10 min in hCMEC/D_3 cells.

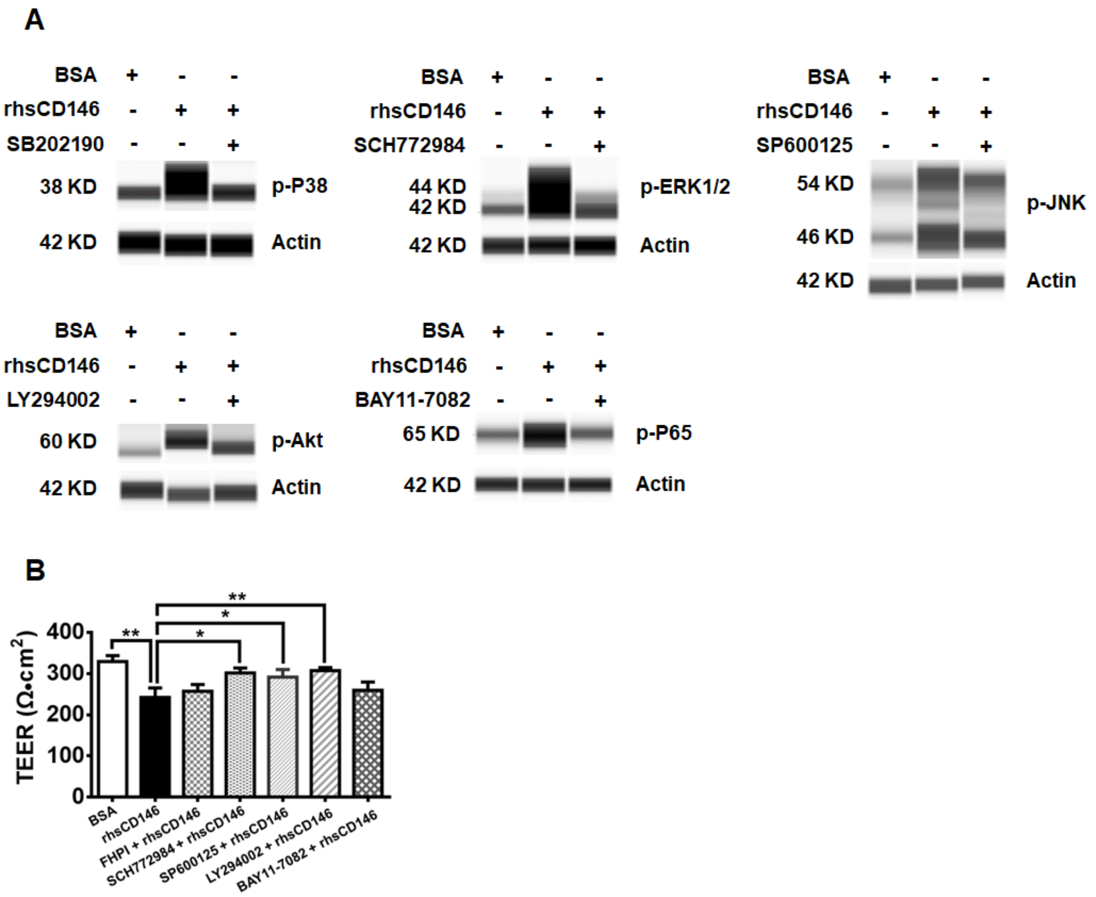

Figure S7. sCD146-induced hyperpermeability of hCMEC/D_3 cells was inhibited by related signaling inhibitors. (A) The abnormal phosphorylation of MAPK, Akt and NF-κB induced by sCD146 in hCMEC/D_3 cells is inhibited by their respective inhibitors. hCMEC/D_3 cells were preincubated with signaling inhibitors 45 min before treatment with 5 μg/mL rhsCD146 for 10 min. The working concentration of signaling inhibitor of p38 (FHPI), JNK (SP600125), and NF-κB (BAY11-7082) is 10 μM, of ERK1/2 (SCH772984) is 2 μM and of Akt (LY294002) is 5 μM. At least three independent assays were performed. (B) MAPK, Akt and NF-κB signaling pathways are involved in sCD146-induced hyperpermeability of hCMEC/D_3 cells. hCMEC/D_3 cells were preincubated with signaling inhibitors 45 min before treatment with 5 μg/mL rhsCD146. The working concentration of signaling inhibitor of p38 (FHPI), JNK (SP600125), and NF-κB (BAY11-7082) is 10 μM, of ERK1/2 (SCH772984) is 2 μM and of Akt (LY294002) is 5 μM. At least three independent assays were performed.

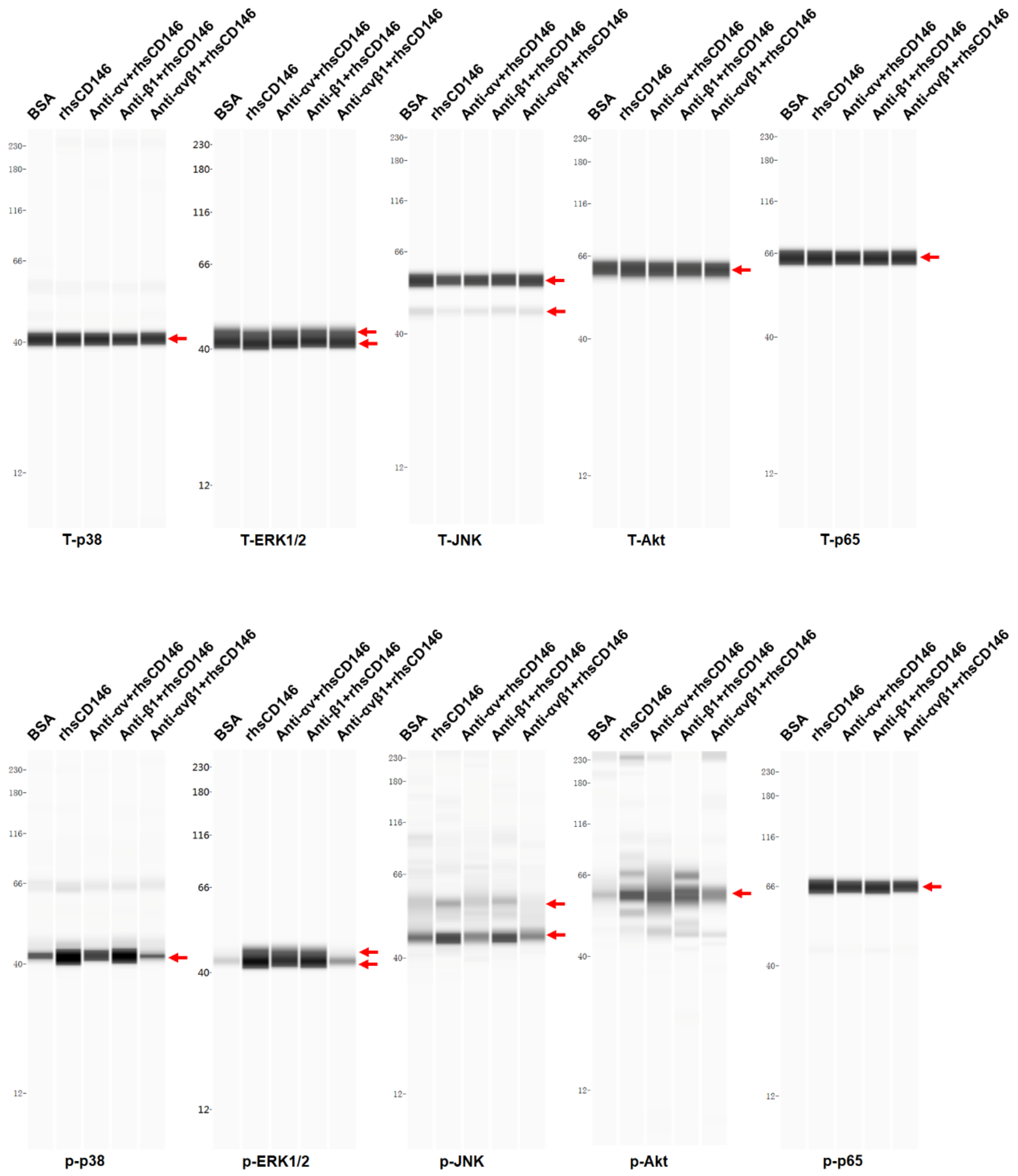

Figure S8. rhsCD146-induced phosphorylation of p38, ERK1/2, JNK, Akt and NF-κB was inhibited by anti-integrin αv and β1 antibodies. hCMEC/D_3 cells were preincubated with 3 μg/mL IgG, anti-integrin αv, anti-integrinβ1 or anti-integrin αvβ1 antibodies for 30 min, and then, 5 μg/mL BSA or rhsCD146 was added to the culture medium and incubated for another 10 min. The cell lysates were harvested for western blot analysis.

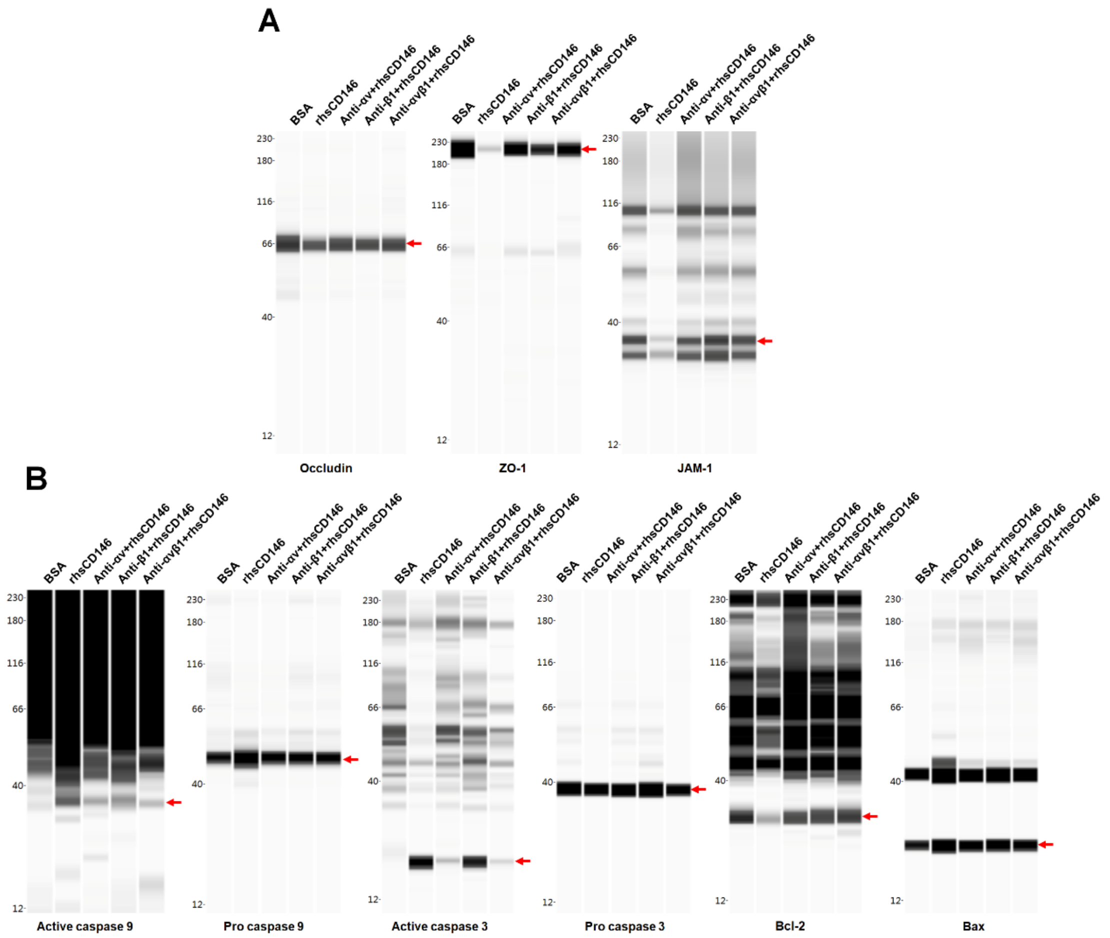

Figure S9. (A) hCMEC/D_3 cells were preincubated with 3 μg/mL IgG, anti-integrin αv or β1 antibody for 30 min and then treated with 5 μg/mL BSA or rhsCD146. TJP expression was verified by western blotting. (B) hCMEC/D_3 cells were preincubated with 3 μg/mL IgG, anti-integrin αv or β1 antibody for 30 min and treated with 5 μg/mL BSA or rhsCD146 for another 12 h. Western blotting was performed to detect the expression of caspase 9, caspase 3, Bcl-2 and Bax.
